# Case report of pulmonary vein isolation in situs inversus totalis

**DOI:** 10.1093/ehjcr/ytae067

**Published:** 2024-02-05

**Authors:** Annina Stauber, Raban Jeger, Omer Dzemali, Andreas Stephan Müller

**Affiliations:** Department of Cardiology, City Hospital Zurich Triemli, Birmensdorferstr. 497, 8063 Zurich, Switzerland; Department of Cardiology, City Hospital Zurich Triemli, Birmensdorferstr. 497, 8063 Zurich, Switzerland; Departement of Cardiac Surgery, City Hospital Zurich Triemli, Birmensdorferstr. 497, 8063 Zurich, Switzerland; Departement of Cardiac Surgery, University Hospital Zurich, Rämistrasse 100, 8091 Zurich, Switzerland; Department of Cardiology, City Hospital Zurich Triemli, Birmensdorferstr. 497, 8063 Zurich, Switzerland

**Keywords:** Atrial fibrillation, Pulmonary vein isolation, Situs inversus totalis, Palpitations, Case report

## Abstract

**Background:**

Situs inversus totalis (SIT) is a rare condition, where all the organs in the body are mirrored. Atrial fibrillation occurs in patients with SIT. We describe the case of pulmonary vein isolation (PVI) in SIT.

**Case summary:**

A patient with atrial fibrillation was referred to our hospital due to palpitations. Diagnosis of atrial fibrillation was made by electrocardiogram. The patient reported to have a SIT that was confirmed. Meticulous preparation was done including a three-dimensional model and radiofrequency PVI was performed successfully. No recurrence of atrial fibrillation was detected until last follow-up 2 years after PVI.

**Discussion:**

Pulmonary vein isolation in SIT can be performed successfully and with excellent long-term result.

**Learning points**

- Pulmonary vein isolation in situs inversus totalis is feasible and can be performed with excellent long-term result, when performed by experienced electrophysiologists.
- Meticulous preparation makes it possible to estimate the degree of difficulty in specific anatomy cases and to optimally prepare for a procedure.

## Introduction

Pulmonary vein isolation (PVI) has become increasingly popular in the last years, and its results have improved with technological advances. However, whether success rates of PVI are similar in patients with atypical anatomy is not known. We describe the case of a patient with situs inversus totalis (SIT) undergoing PVI. In SIT, all the thoracic and abdominal organs are mirrored.

## Summary figure

**Table:** 

1999	First manifestation of palpitations and chest pain
01/2021	Electrocardiogram (ECG) and transthoracic echocardiography confirmed the situs inversus reported by the patient
03/2021	Coronary angiography ruled out coronary heart disease
03/2021	Computed tomography confirmed diagnosis of situs inversus totalis
03/2021	ECG revealed paroxysmal atrial fibrillation
06/2021	Pulmonary vein isolation for atrial fibrillation was performed
06/2023	Follow-up including long-term ECG demonstrating stable sinus rhythm

## Case summary

**Figure 1:**
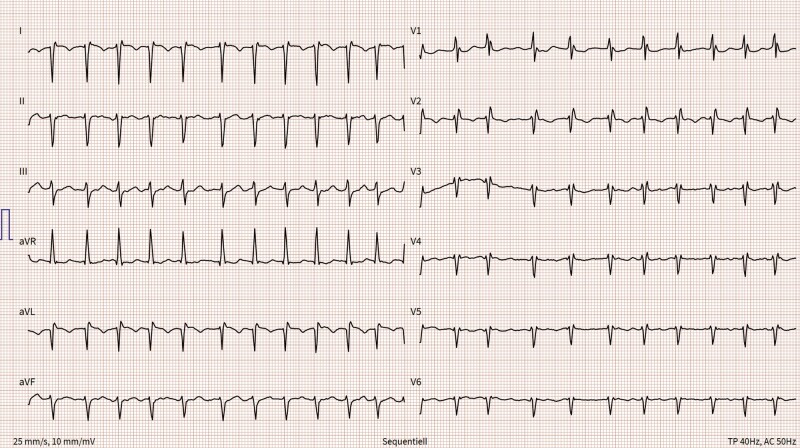
Electrocardiogram in dextrocardia showing atrial fibrillation: right axis deviation with positive QRS complexes in aVR and negative QRS complexes in the lead I and aVL. R waves continuously decrease from V1 to V6.

We report the case of a 68-years old, otherwise healthy, female patient being referred to our hospital due to chest pain and palpitations. At the first visit in the outpatient clinic, vital parameters were within normal range and she was cardiopulmonary compensated. Twelve lead electrocardiogram (ECG) showed signs of dextrocardia and transthoracic echocardiography was challenging but supported the diagnosis of situs inversus, which was reported by the patient. The patient was admitted to hospital for coronary angiography, which ruled out coronary heart disease. A computed tomography (CT) was performed, and a complete situs inversus totalis was documented, with the heart and all the other organs mirrored. No cardiac malformation was found. During this hospitalization, symptomatic tachycardic atrial fibrillation was documented on ECG (*Figure 1*). The existing medication with bisoprolol that was initially started with 2.5 mg for palpitations was increased to 10 mg daily and rivaroxoban 20 mg daily was administered. However, she remained symptomatic. In discussion with the patient and the team, the patient refused to take antiarrhythmic drugs because of the potential side effects and together the decision to perform the PVI was made.

### Preparation before procedure

**Figure 2:**
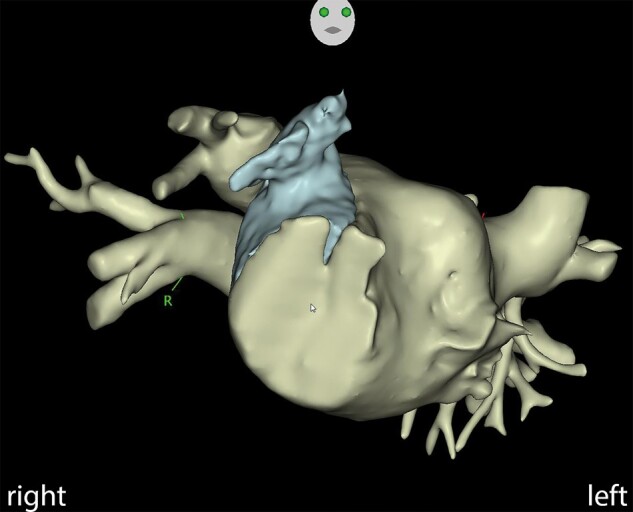
3D reconstruction of cardiac CT, showing the systemic atrium and atrial appendage (RAO view).

We studied the mirrored anatomy on CT in detail. The CT was segmented in the CARTOSEG TM® system (Biosense Webster, NJ, USA) (*Figure 2*). Both improved our understanding of anatomy. However, understanding the anatomy is the basic requirement, but precise control and guidance of the catheters, sheaths, and needles are also essential for the successful performance of a procedure. It seemed that this could be best prepared by a three-dimensional (3D) model and therefore we built one. Every important step of the procedure was performed in this model prior to the procedure using similar materials to those used in the procedure.

### Ablation procedure

**Figure 3:**
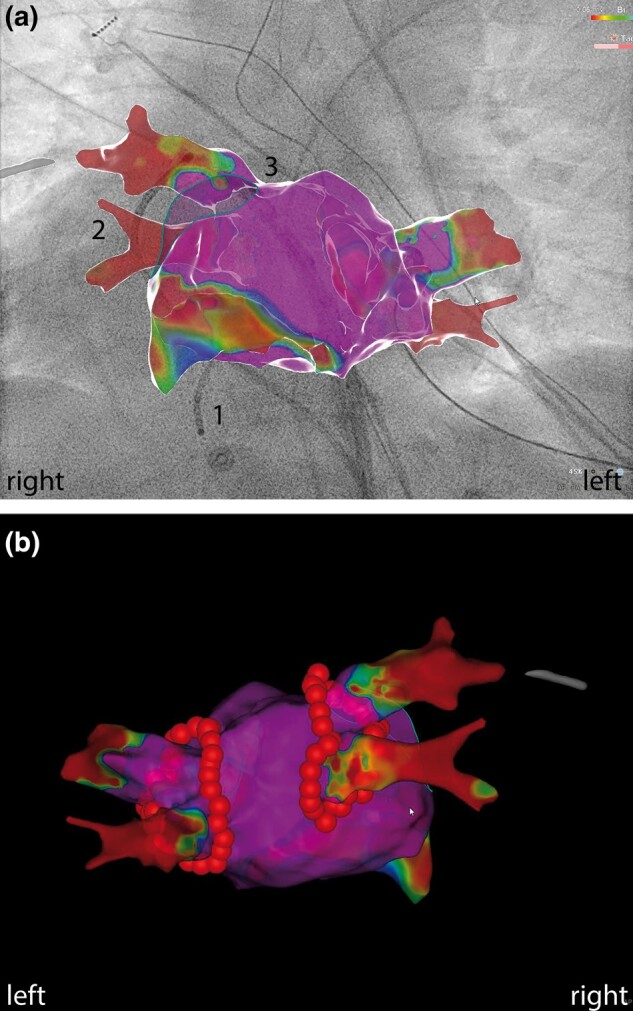
(*A*) CARTO 3D voltage map with normal myocardium, (1) catheter in subpulmonary ventricular apex, (2) coronary sinus catheter, (3) ablation catheter (AP view). (*B*) CARTO 3D map, successful ablation with ablation points (PA view).

**Figure 4:**
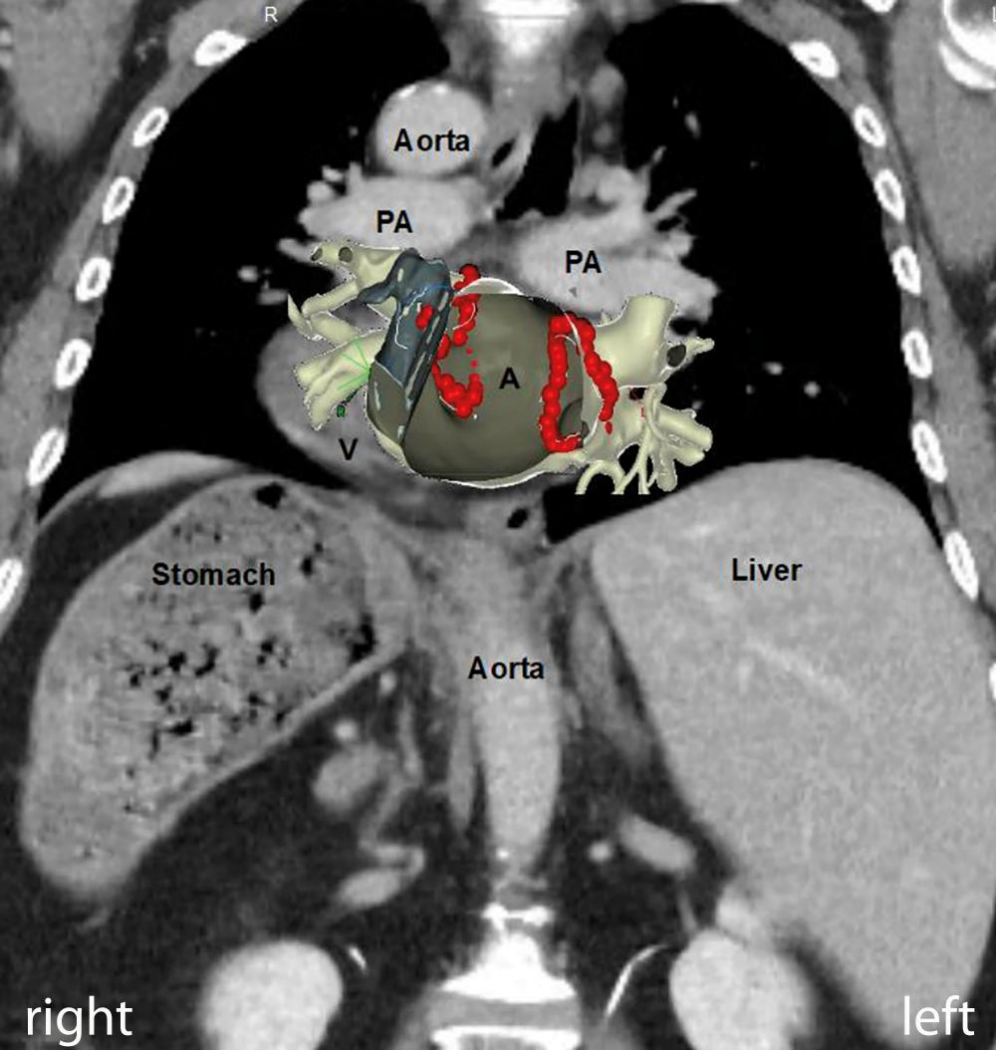
CT with overlaying Carto geometry. A, atrium; V, ventricle; PA, pulmonary artery.

Ablation was performed under general anaesthesia. The ultrasound-assisted venous access was chosen via right groin puncture. A quadripolar catheter was placed into the subpulmonary ventricle, and a decapolar catheter was placed into the coronary sinus. A guide wire was positioned into the superior vena cava, and a non-steerable long sheath (SL0, Abbott, St. Paul, MN, USA) was placed over the wire into the superior vena cava. The wire was exchanged with a Brockenbrough™ needle (BRK-1, Abbott, St. Paul, MN, USA). The sheath-needle system was retracted at an 8 o’clock position until the second ‘jump’. The position of the needle in the septum was checked with an X-ray [right anterior oblique (RAO) 47° and left anterior oblique (LAO) 39°] and transoesophageal echocardiography (TEE, view rotation 83°). Transseptal puncture (TSP) was performed on the first attempt without complication (see Supplementary material online, *Videos S1* and *S2*). The guide wire was advanced to the lateral superior pulmonary vein above the atrial appendage. The non-steerable sheath was exchanged with a steerable sheath (Agilis, Abbott, St. Paul, MN, USA). The non-steerable sheath was placed in the subpulmonary atrium via a second guide wire. Next, the ablation catheter was introduced into the systemic atrium in a two-over-one method. A PentaRay®NAV catheter (Biosense Webster, NJ, USA) was introduced via the steerable sheath into the systemic atrium. The 3D map was acquired with the PentaRay®NAV catheter (Biosense Webster, NJ, USA). The voltage map showed normal voltage myocardium (*Figure 3A*). The sheath-catheter combination was changed for ablation, and ablation was performed with the THERMOCOOL SMARTTOUCH® SF Catheter (Biosense Webster, NJ, USA) in the steerable sheath (Agilis, Abbott, St. Paul, MN, USA). The wide antral cirumferential ablation was performed with 30 W at the posterior wall and 40 W at the anterior wall (target ablation index 400 posterior, 550 anterior). First pass isolation was achieved on both sides, confirmed by entrance and exit block (*Figures 3B* and *4*). Procedure time was 132 min, fluoroscopy time 7.9 min, X-ray dose (dose area product) 83.4 cGy cm^2^, and total ablation time 31 min.

### Follow-up

No atrial fibrillation symptoms were reported until the most recent control 2 years after the procedure. After six weeks, a 48 h long-term ECG and after six months and 2 years a 72 h long-term ECG were performed and none of them showed atrial fibrillation recurrence.

## Discussion

### Electrocardiogram

SIT is a rare condition and sometimes this condition can remain undetected for decades. A first hint for SIT can be found on ECG due to the dextrocardia.^1^ Electrocardiogram in dextrocardia shows a right axis deviation, positive P and QRS in lead aVR, and negative P and QRS in lead I and aVL, and R waves are continuously decreasing from V1 to V6. If the leads are positioned mirrored, the ECG changes can be unmasked. An important differential diagnosis is the dextroposition of the heart, where the heart is shifted to the right side but not mirrored (e.g. in case of right-sided pneumothorax). In that case, ECG cannot be reversed by positioning the leads mirrored.

### Pulmonary vein isolation in situs inversus totalis

PVI is one of the standard procedures in electrophysiology. In special anatomical situations such as SIT, challenges can be mastered by meticulous preparation. In this case, CT helped to understand detailed anatomy. CT and transthoracic echocardiogram were able to exclude additional congenital heart disease, which are more prevalent in SIT.^2^ We created a 3D model prior to the procedure, whereby the mirrored anatomy was studied and understood in detail. We considered performing the procedure from the left-sided groin and mirroring the X-ray projections, but this idea was discarded given the additional unfamiliarity.

TSP turned out to be the most crucial step of the procedure. The needle-sheath system for TSP was held in an 8 o’clock position as described in literature, mirroring the 4 o’clock position in patients without SIT.^3^ It was guided by X-ray as usually done at our centre and additionally TEE. Good preparation with deep insertion of a coronary sinus catheter into the distal coronary sinus helped to visualize the anatomy of the atrioventricular groove radiologically and to guide transseptal access. We found that TEE contributed to additional safety in this specific case, because the correct position in X-ray could be confirmed by a second image modality. Transoesophageal echocardiography rotation in our case was 83°, somehow mirroring the standard 135°. Intracardiac echocardiography (ICE) could have been another modality to use, but our experience with ICE guided TSP is limited. For mapping and ablation the same catheters and sheets as we usually use for routine PVIs were used. Also here, movements had to be performed mirrored and every movement had to be considered carefully beforehand. 3D model was very useful to learn these mirrored movements.

Screening the literature about PVI in SIT, minimal experience is found because SIT is a rare condition (∼1:10 000).^2^ Due to its rare nature and lack of evidence, there is no specific advice how to treat patients with atrial fibrillation and SIT in the ESC guidelines.^4^ To the best of our knowledge, no severe complication in PVI in SIT is documented in literature. It is essential to assess the risk–benefit individually for every patient since a long procedure duration or high radiation times and doses were found in some reports.^5–8^ In our case, the long-term result was excellent, no complication occurred and the procedural data (X-ray time, X-ray dose, procedure time, ablation time) were similar to patients with normal anatomy.^9–11^

## Conclusion

To conclude, PVI in SIT is feasible and can be performed successfully without complication, with excellent long-term result and with procedural data comparable to PVI with normal anatomy. However, meticulous preparation is crucial.

## Supplementary Material

ytae067_Supplementary_Data

## Data Availability

The data underlying this article cannot be shared publicly due to privacy reasons. The data will be shared on reasonable request to the corresponding author.

## Lead author biography

Annina Stauber has completed her medical education and cardiology training in Switzerland. She was an electrophysiology Fellow 2017–2018 in Heart Center Leipzig, Germany. Currently, she works for City Hospital Zurich Triemli in Switzerland.

## References

[1] Golzarian H, Widmer MB, Mughal S, Patel SM. ECG challenge: beating at the right place at the right time. Eur Heart J Case Rep 2023;7:ytad094. 10.1093/ehjcr/ytad094 PMC9991043 36895296

[2] Eitler K, Bibok A, Telkes G. Situs inversus totalis: a clinical review. Int J Gen Med 2022;15:2437–2449. 35264880 10.2147/IJGM.S295444 PMC8901252

[3] Zhao X, Su X, Long DY, Sang CH, Bai R, Tang RB, et al Catheter ablation of atrial fibrillation in situs inversus dextrocardia: challenge, improved procedure, outcomes, and literature review. Pacing Clin Electrophysiol 2021;44:293–305. 33372281 10.1111/pace.14144

[4] Hindricks G, Potpara T, Dagres N, Arbelo E, Bax JJ, Blomström-Lundqvist C, et al 2020 ESC guidelines for the diagnosis and management of atrial fibrillation developed in collaboration with the European Association for Cardio-Thoracic Surgery (EACTS): the task force for the diagnosis and management of atrial fibrillation of the European Society of Cardiology (ESC) developed with the special contribution of the European Heart Rhythm Association (EHRA) of the ESC. Eur Heart J 2021;42:373–498. 32860505 10.1093/eurheartj/ehaa612

[5] Del Greco M, Marini M, Centonze M, Disertori M. Atrial fibrillation ablation procedure using electroanatomic reconstruction of the right and left atrium in a patient affected by dextrocardia. Europace 2009;11:1399–1400. 19654126 10.1093/europace/eup209

[6] Xue ZM, Sang CH, Dong JZ, Ma CS. Catheter ablation of persistent atrial fibrillation in a patient with dextrocardia. Chin Med J (Engl) 2012;125:1839–1840. 22800910

[7] Chong E, Chang SL, Chen SA. Pulmonary vein isolation in a patient with dextrocardia. Europace 2012;14:1725. 23002200 10.1093/europace/eus325

[8] Okajima K, Nakanishi T, Ichibori H, Shirai T, Kadotani M, Shimizu H, et al Trans-aortic pulmonary vein isolation using magnetic navigation system for paroxysmal atrial fibrillation in a patient with dextrocardia, situs inversus, and inferior vena cava continuity with azygos vein. J Arrhythm 2018;34:583–585. 30327707 10.1002/joa3.12096 PMC6174455

[9] Stauber A, Kornej J, Sepehri Shamloo A, Dinov B, Bacevicius J, Dagres N, et al Impact of single versus double transseptal puncture on outcome and complications in pulmonary vein isolation procedures. Cardiol J 2021;28:671–677. 32207839 10.5603/CJ.a2020.0037 PMC8428934

[10] Mulder MJ, Kemme MJB, Allaart CP. Radiofrequency ablation to achieve durable pulmonary vein isolation. Europace 2022;24:874–886. 34964469 10.1093/europace/euab279

[11] Holmqvist F, Kesek M, Englund A, Blomström-Lundqvist C, Karlsson LO, Kennebäck G, et al A decade of catheter ablation of cardiac arrhythmias in Sweden: ablation practices and outcomes. Eur Heart J 2019;40:820–830. 30452631 10.1093/eurheartj/ehy709 PMC6403459

